# Lost Branches on the Tree of Life

**DOI:** 10.1371/journal.pbio.1001636

**Published:** 2013-09-03

**Authors:** Bryan T. Drew, Romina Gazis, Patricia Cabezas, Kristen S. Swithers, Jiabin Deng, Roseana Rodriguez, Laura A. Katz, Keith A. Crandall, David S. Hibbett, Douglas E. Soltis

**Affiliations:** 1 University of Florida, Gainesville, Florida, United States of America; 2 Clark University, Worcester, Massachusetts, United States of America; 3 Brigham Young University, Provo, Utah, United States of America; 4 George Washington University, Washington, DC, United States of America; 5 Smith College, Northampton, Massachusetts, United States of America; 6 Florida Museum of Natural History, Gainesville, Florida, United States of America

## Abstract

Failure to archive published data can impede reproducibility and inhibit downstream synthesis. Alarmingly, we estimate that ∼70% of existing DNA sequence alignments/phylogenetic trees, representing much of the underpinning of modern phylogenetic analysis, are no longer accessible. The evolutionary biology community needs to adopt policies ensuring that data are publicly archived upon publication.

## Introduction

Given that reproducibility is a pillar of scientific research, the preservation of scientific knowledge (underlying data) is of paramount importance. The standard of reproducibility can be evaluated based on criteria of methodological rigor and legitimacy, which is sometimes used to distinguish “hard” from “soft” sciences. In phylogenetics, a discipline that routinely uses DNA sequences to build trees reflecting organismal relationships, the scale of data collection and the complexity of analytical software have both increased dramatically during the past decade. Consequently, the ability to navigate publications and reproduce analyses is more challenging than ever. When DNA sequencing was initially employed in systematics during the late 1980s, there was some reluctance to deposit nucleotide sequences in open repositories such as GenBank [1]. This ultimately changed when high-impact journals (e.g., *Proceedings of the National Academy of Sciences*, *Nature*, *Science*) began requiring GenBank submission as a prerequisite for publication [1],[2]; now virtually every evolutionary biology journal observes this requirement (but see [3]).

Until recently, uploading sequences to GenBank (or EMBL) was generally considered sufficient to ensure reproducibility of phylogenetic studies using DNA sequence data. Increasingly, however, the systematics community is realizing that archiving raw DNA sequences is not adequate, and that the underlying alignments of DNA sequences as well as the resulting phylogenetic trees are pivotal for reproducibility, comparative purposes, meta-analyses, and ultimately synthesis. Indeed, there has been a growing clamor for journals to adopt and enforce more rigorous data archiving practices across diverse disciplines [4]–[8]. As a result, about 35 evolutionary journals [5],[9] have adopted policies to encourage or require authors to upload alignments, phylogenetic trees, and other files requisite for study reproducibility [5] to TreeBASE (http://treebase.org/) and/or other public repositories such as Dryad (http://datadryad.org). Unfortunately, enforcement of such data deposition policies is generally lax, and most journals in systematics and evolution still do not require DNA sequence alignment or tree deposition. As a result, the alignments and trees underlying most published papers in systematics/phylogenetics and evolutionary biology remain inaccessible to the scientific community at large [8],[10].

## Scope of the Problem

As DNA sequencing has become easier, faster, and cheaper, and as scientists have come to realize that phylogenies inform diverse areas of inquiry, phylogenetic trees have permeated virtually every facet of biology, including disparate subdisciplines such as medicine (e.g., [11],[12]), climate change research (e.g., [13],[14]), organismal evolution (e.g., [15]), conservation efforts (e.g., [16]), and linguistics (e.g., [17]). In building phylogenetic trees, researchers implicitly acknowledge that alignments and trees are important. However, archiving these data has been largely ignored, perhaps because researchers have considered the actual raw sequence data as the sole information necessary to replicate a phylogenetic study, while alignments and phylogenetic trees have been treated as the resulting outcome from sequence data analyses. The latter view of alignments and trees is certainly correct, but the underlying sequence alignments and associated trees should also be recognized as crucial data in their own right. The increasing use of published trees and the underlying sequence alignments as the framework for evolutionary inference and other subsequent downstream hypothesis testing dictates, however, that alignments and trees *are* data and need to be archived with a diligence on par with raw sequence data.

The call for ensuring reproducibility and data sharing in systematics is not new. The fundamental importance of archiving scientific datasets across numerous subdisciplines including climate change research, evolutionary biology, and medicine has received increasing attention over the past five years [5]–[8],[10],[18]–[22]. Several of these studies have examined the proportion of publications that archived data in a manner that affords public access [6],[8],[18], and all concluded that we have entered an age in which scientific journals should require and enforce data archiving policies.

Some researchers, including [23] for psychology and [4] for medical research, have taken the next step and have contacted authors directly when data of interest have not been available, which highlighted an additional problem. These workers found that data are not easily obtained via direct author contact. More recently, Stoltzfus et al. [8] examined deposition practices within the molecular systematic community, and estimated alignment/tree deposition rates to be remarkably low (∼4%). Stolzfus et al. [8] focused on only two journals (*American Journal of Botany* and *Evolution*), and searched literature over just a 2-year period (2010–2011). Although the study of Stolzfus et al. [8] represents a good first step, no analysis has attempted to evaluate how often alignments/trees are deposited over a broad range of evolutionary biology journals that span organismal diversity representing the tree of life, or how archiving tendencies have changed over time.

In the process of gathering data to build the first tree of life for all ∼1.9 million named species (the Open Tree of Life Project; http://opentreeoflife.org), we examined 7,539 peer-reviewed papers to evaluate data depositional practices of foundational DNA sequence alignments and phylogenetic trees by the systematic community between 2000 and 2012. Our broad survey of the literature covered animals, fungi, seed plants, microbial eukaryotes, archaea, and bacteria, and included publications from more than 100 journals (see Tables S1, S2, S3, S4). To assess the rigor of data that were deposited in a public archive, we also examined the quality (e.g., Did deposited trees match publication figure(s)? Were there branch lengths in deposited trees?) of ca. 350 files deposited in TreeBASE (described in Text S1). Additionally, we attempted to acquire data by randomly contacting 375 authors directly (see Text S1 and Table S4). Furthermore, to evaluate depositional practices of other data critical for study replication, we surveyed 100 randomly selected publications that implemented the popular evolutionary analysis package BEAST (Bayesian Evolutionary Analysis Sampling Trees [24]; 4,153 citations as of 7-17-2013), which is widely used to obtain divergence times and phylogenies that are used to test hypotheses and draw conclusions regarding broad biological questions (e.g., phylogeography, lineage origins).

**Figure 1:**
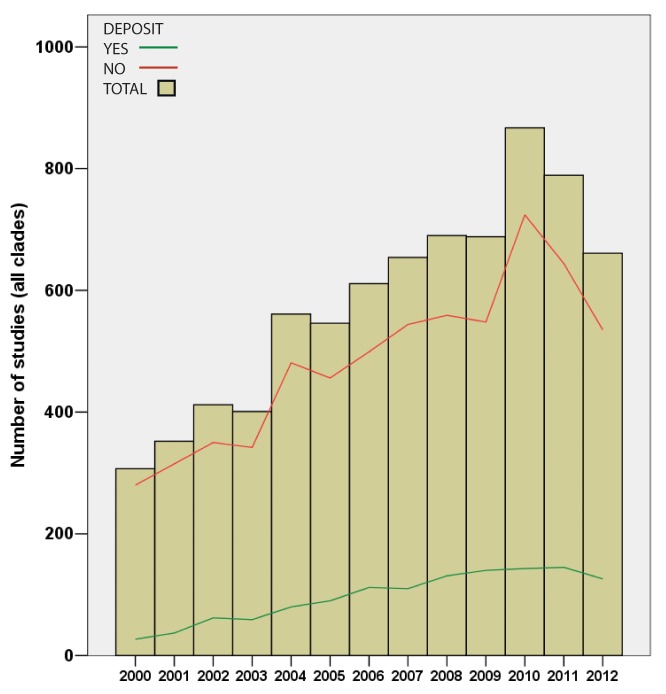
Overview of total number of publications surveyed from animal, fungus, seed plant, microbial eukaryote, archaea, and bacteria literature (indicated in red), and the number of those publications that archived their trees and alignments in either Dryad or TreeBASE (indicated in green).

**Figure 2:**
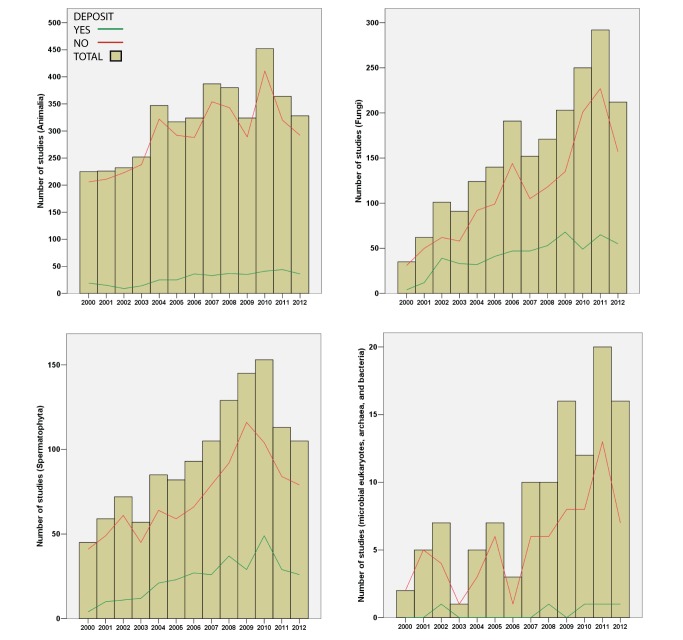
Graphs showing results of publication surveys from four disparate domains of life: (1) animals; (2) fungi; (3) spermatophytes; and (4) microbial eukaryotes, archaea, and bacteria. Red lines indicate total number of publications surveyed and green lines indicate the number of those publications that made their data accessible in either Dryad or TreeBASE.

Surprisingly, only 16.7%, 1,262 from a total of 7,539 publications surveyed, provided accessible alignments/trees (Figures 1 and 2). Our attempts to obtain datasets directly from authors were only 16% successful (61/375; see Table S4), and we estimate that approximately 70% of existing alignments/trees are no longer accessible. Thus, we conclude that most of the underlying sequence alignments and phylogenetic trees produced by the systematic community during the past several decades are essentially lost, accessible only as static figures in a published journal article with no capacity for subsequent manipulation. Furthermore, when data are deposited, they are often incomplete (e.g., what characters were excluded, accepted taxon names; see Text S1 and Figure S1). Our survey of publications that implemented BEAST revealed that only 11 out of 100 (11%) examined studies provided access to the underlying xml input file, which is critical for reproducing BEAST results. Although funding agencies often require all data to be accessible from funded publications, our results reveal this is more the exception than the rule.

## Failure on a Massive Scale

Our findings indicate that while some journals (e.g., *Evolution*, *Nature*, *PLOS Biology*, *Systematic Biology*) currently require nucleotide sequence alignments, associated tree files, and other relevant data to be deposited in public repositories, most journals do not have these requirements; resultantly, the systematics community is doing a poor job of making the actual datasets available. More troublesome perhaps is that the situation has barely improved over the 12 years covered in this study (Figures 1 and 2). In addition, when data are deposited, they often do not include critical information such as what was actually included in data alignments (e.g., what characters were excluded, full taxon names; see Table S1 and Figure S1). Without accurate details describing how alignments were implemented, it is difficult or perhaps impossible to faithfully reproduce the study results. Additionally, parameters for the program BEAST are rarely made available for scrutiny. Lastly, in many cases when data were not deposited to TreeBASE, the authors indicated that the data could be obtained directly from them; however, our survey indicates this is typically not the case (only ∼40% of authors even respond, and of these only a small percent actually provide the requested data)—hence, many alignments and analysis parameters seem to be lost forever.

To some extent it is understandable why trees and alignments are not always deposited in available databases [8]. Though some funding sources (e.g., NSF) currently require a data management plan for grant proposals, explicit requirements regarding postpublication data archiving are lacking, and there is little if any postfunding oversight into data archiving practices. Also, authors may be leery of making their data public after investing a great deal of time and money in their compilation, fearing their data will be quickly reused without an offer of co-authorship or even an acknowledgment. For example, it is common practice to obtain sequence data from GenBank and refer to accession numbers without citing the originating paper. However, in today's academic world where citations are paramount, accession numbers provide no direct indication of the original authors' contribution. This is an ethical question best dealt with elsewhere, but nonetheless extremely important. Suffice it to say, if large parts of previously published phylogenies are reused, the original source(s) should at least be cited. Additionally, after the arduous process authors face in preparing and uploading manuscripts, the last thing they want to do is struggle with still another upload, especially when it is optional. Thus, databases that house information (e.g., TreeBASE, Dryad) must ensure that the process of entering alignments, trees, and other relevant data is user-friendly and not time consuming. Dryad currently accepts various file formats and is straightforward to use, while TreeBASE archives trees and alignments, requires nexus files, and is more difficult to navigate (especially the first time through). Although TreeBASE has become much easier to use during the past 2 years, it can still be time consuming. Also, unlike GenBank and Dryad, TreeBASE does not currently make data publicly available automatically upon publication; authors often upload data into TreeBASE for reviewers, but do not subsequently make data available for public viewing upon manuscript acceptance or publication. Repositories that permit easy data uploading will help encourage authors to view these databases as a way to make their data permanently available (and cited) as opposed to being yet another hurdle to overcome on the road to publication. Lastly, we stress that authors associated with the writing of this paper also have published studies that do not contain external links to our alignment data; hence, we are not pointing fingers, but rather elucidating a widespread problem (indeed, a culture) and suggesting solutions (a cultural revolution). We are in a digital age, and our data archiving practices need to keep pace with our ability to generate data, DNA sequence alignments, and phylogenetic trees. For our science to have the broadest impacts, we need to move beyond the notion that deposition of raw sequences is sufficient and realize that our phylogenetic estimates are of broad value and utility and should be provided to potential users in a format other than an image in a static pdf file.

## Moving Forward

The systematics community needs to substantially improve efforts to ensure that data (e.g., trees, alignments, BEAST xml files) are available to others in the scientific community. A logical first step in ensuring that alignment and tree files are deposited in one of the commonly used databases is for scientific journals to require and enforce such depositions. A new “data deposition” metric, such as number of genes×number of taxa/number of publications, could be devised to confer prestige to well-published and well-archived authors. More simply, a single metric such as number of publications with archived data could be a standard CV item. These depositions should also include program input files (e.g., xml files) for popular programs such as BEAST, as well as any other relevant information needed to replicate the study. Optimally, all peer-reviewed journals that publish phylogenetic datasets should require deposition (and activation for public access) of alignments and trees prior to publication, and these trees and alignments will include the same characters and taxa (and taxon names) as in the published study. Funding agencies can (and should) facilitate the process of making data matrices and phylogenetic trees publicly available by explicitly requiring data archiving as part of data management plans. In addition, a summary of data archiving should become a mandatory feature of annual and final project reports to funding agencies. Archiving efforts could be quantified and rewarded by reporting previously archived data as part of new grant proposals.

Perhaps more importantly, we call for a shift in thinking among all evolutionary biologists who rely on the power of phylogenetics to test hypotheses and make inferences. It is crucial for this broad discipline to consider the alignments and phylogenies themselves as key data that require appropriate storage for study reproducibility and data integration. The sheer volume of sequence data that are continually generated and processed, along with the myriad of programs available for data analysis, dictate the urgent need to adopt policies requiring public archiving of alignments and trees as a requirement of publication. The biological community has lost most of the alignments and trees underlying the numerous phylogenetic analyses conducted over the past several decades—we should strive to do much better in the years ahead. Ideally, we will move forward as a community and require ourselves to deposit our alignments, phylogenies, and other relevant data as a matter of course.

## Supporting Information

Figure S1 **Quality survey from 344 publications that did have data publicly available on TreeBASE (see Text S1).**
(TIF)

Table S1 **List of specialized journals (see definition in Text S1) examined here.**
(DOCX)

Table S2 **List of nonspecialized, broad audience journals (see definition in Text S1) examined here.**
(DOC)

Table S3 **List of 74 specialized journals surveyed for seed plant phylogenies.**
(DOC)

Table S4 **Corresponding author's response to emails (2) requesting alignments and trees from previously published study.** Number in parenthesis subtending organismal group represents number of authors contacted.
(DOCX)

Text S1 **Description of methods used for this study.**
(DOC)
