# Supplementary material for: Lost Branches on the Tree of Life
Source: PLoS Biol. 2013 Sep 3;11(9):e1001636. doi: 10.1371/journal.pbio.1001636 (PMC3760775; doi:10.1371/journal.pbio.1001636)
Supplement: Table S1 — List of specialized journals (see definition in Text S1) examined here. (DOCX) [file pbio.1001636.s002.docx]

Table S1. List of specialized journals (see definition in Text S1) examined here.

| **FUNGI** | **SPERMATOPHYTA** | **ANIMALIA** | **MICROBIAL EUKARYOTES, ARCHAEA, and BACTERIA** |
| --- | --- | --- | --- |
| *Cryptogamie (Mycologie)* | *Annals of Botany* | *Cladistics* | *The Journal Of Eukaryotic Microbiology* |
| *Fungal Biology* | *Australian Systematic Botany* | *COPEIA* | *Archives Of Microbiology* |
| *Fungal Diversity* | *Aliso* | *Invertebrate Systematics* | *BMC Evolutionary Biology* |
| *Fungal Ecology* | *Annals of the Missouri Botanical Garden* | *Journal of Arachnology* | *Eukaryotic Cell* |
| *Mycologia* | *American Journal of Botany* | *Journal of Crustacean Biology* | *European Journal Of Protistology* |
| *Mycological Progress* | *Biological Journal of the Linnean Society* | *Journal of Herpetology* | *Extremophiles: Life Under Extreme Conditions* |
| *Mycological Research* | *Botanical Journal of the Linnean Society* | *Journal of Mammalogy* | *Genomics* |
| *Mycorrhiza* | *Cladistics* | *Journal of Zoological Systematics and Evolutionary Research* | *International Journal Of Systematic And Evolutionary Microbiology* |
| *Mycoscience* | *International Journal of Plant Sciences* | *Organisms Diversity & Evolution* | *International Journal Of Systematic Bacteriology* |
| *Mycotaxon* | *Journal of Plant Research* | *Systematic Entomology* | *Protist* |
| *New Phytologist* | *Plant Systematics and Evolution* | *Zoologica Scripta* | *The Journal Of Eukaryotic Microbiology* |
| *Nova Hedwigia* | *Plant Biology* | *Zoological Journal Of The Linnean Society* |  |
| *Persoonia* | *Systematic Botany* | *Zoosystematics And Evolution* |  |
| *Phytopathology* | *Taxon* | *Zootaxa* |  |
| *Studies in Mycology* | Miscellaneous (74 different journals with < 10 articles surveyed; see Table S3) |  |  |
| *Sydowia* |  |  |  |
| *Taxon* |  |  |  |
| *The lichenologist* |  |  |  |
