# Supplementary material for: Lost Branches on the Tree of Life
Source: PLoS Biol. 2013 Sep 3;11(9):e1001636. doi: 10.1371/journal.pbio.1001636 (PMC3760775; doi:10.1371/journal.pbio.1001636)
Supplement: Table S2 — List of nonspecialized, broad audience journals (see definition in Text S1) examined here. (DOC) [file pbio.1001636.s003.doc]

Table S2. List of non-specialized, broad audience journals (see definition in Text S1) examined here.

| *Evolution* | *Molecular Phylogenetics and Evolution* | *Proceedings of the National Academy of Sciences* |
| --- | --- | --- |
| *Molecular Biology and Evolution* | *Nature* | *Science* |
| *Molecular Ecology* | *PLoS ONE* | *Systematic Biology* |
