# Supplementary material for: Lost Branches on the Tree of Life
Source: PLoS Biol. 2013 Sep 3;11(9):e1001636. doi: 10.1371/journal.pbio.1001636 (PMC3760775; doi:10.1371/journal.pbio.1001636)
Supplement: Table S3 — List of 74 specialized journals surveyed for seed plant phylogenies. (DOC) [file pbio.1001636.s004.doc]

Table S3. List of 74 specialized journals surveyed for seed plant phylogenies.

| *Acta Phytotaxonomica Sinica* | *Evolutionary Bioinformatics* | *Perspectives in Plant Ecology, Evolution, & Systematics* |
| --- | --- | --- |
| *Acta Botany Yunnanica* | *Folia Geobotanica* | *Phytochemistry* |
| *American Naturalist* | *Flora* | *Phytotaxa* |
| *Antarctic Science* | *Gayana Botany* | *Plant Biosystems* |
| *Applied Genetics* | *Genome Biology* | *Plant Ecology & Diversity* |
| *Aquatic Botany* | *Global Change Biology* | *Plant Ecology & Evolution* |
| *Århus* | *Haseltonia* | *Plant Species Biology* |
| *Australian Journal of Botany* | *Harvard Papers in Botany* | *PLoS One* |
| *Biochemical Systematics & Ecology* | *Journal of Botanical Research Institute of Texas* | *Proceedings Biological Society Washington* |
| *Biodiversity & Ecology* | *Journal of Molecular Evolution* | *Proceedings of the California Academy of Sciences* |
| *Biodiversity Resources & Conservation* | *Journal of Plant Sciences* | *Proceedings of the Royal Society B* |
| *Blumea* | *Journal of the Torreya Botanical Society* | *Review of Palaeobotany and Palynology* |
| *Biochemical Sytematics and Evolution* | *Journal of Tropical & Subtropical Botany* | *Revist. Acad. Colombiana Cienc. Exact. Físic. Nat* |
| *BMC Evolution Biology* | *Journal of Biogeography* | *Schumannia* |
| *BMC Plant Biology* | *Journal of Systematics & Evolution* | *Senckenbergiana biologica* |
| *Botanical Bulletin of Academia Sinica* | *Kew Bulletin* | *Smithsonian Contributions to Botany* |
| *Botanische Jahrbücher* | *Korean Journal of Plant Taxonomy* | *South African Journal of Botany* |
| *Botany* | *Lankesteriana* | *Systematics & Biodiversity* |
| *Bothalia* | *Lindleyana* | *Systematics & Geography of Plants* |
| *Brittonia* | *Molecular biology reports* | *Telopea* |
| *Canadian Journal of Botany* | *Molecular Ecology* | *The Gardens’ Bulletin, Singapore* |
| *China Botany Studies* | *New Phytologist* | *Trends in Plant Sciences* |
| *Current Biology* | *New Zealand Journal of Botany* | *Turkish Journal of Botany* |
| *Diversity & Distributions* | *Nordic Journal of Botany* | *Willdenowia* |
| *Edinburgh Journal of Botany* | *Organisms Diversity & Evolution* |  |
