# Supplementary material for: Lost Branches on the Tree of Life
Source: PLoS Biol. 2013 Sep 3;11(9):e1001636. doi: 10.1371/journal.pbio.1001636 (PMC3760775; doi:10.1371/journal.pbio.1001636)
Supplement: Text S1 — Description of methods used for this study. (DOC) [file pbio.1001636.s006.doc]

**Methods**

As we gathered phylogenetic trees and datasets for the Open Tree of Life, we simultaneously evaluated current practices in publishing and archiving alignments and phylogenetic trees. For this task we conducted a literature survey to identify peer-reviewed studies that used molecular data to reconstruct phylogenetic relationships of organismal clades covering animals, fungi, microbial eukaryotes, archaea, bacteria, and seed plants. Nine non-specialized (broad audience) journals (see supplemental data Table S2) and ~100 specialized (i.e., fungi specific, plant specific; Table S1) journals, published between 2000 and 2012, were examined (see Tables S1-S3). For each journal and for each taxonomic category, we determined the total number of phylogenetic studies deposited in the repositories TreeBASE and Dryad. Our basic strategy was to compile a comprehensive database of studies that used phylogenetic analyses (not restricted to systematic work) as part of their methodology; we then determined if the authors deposited their sequence alignments and tree files in either of the two aforementioned databases.

The Open Tree of Life project encompasses ten collaborative institutions including four organismal research laboratories; as a result, each working group adopted different data retrieval approaches that seemed most appropriate for that lineage. For animals, the search was conducted using the Zoological Record online resource (<http://wokinfo.com/products_tools/specialized/zr/>) and publications that matched “phylogen” in the title, abstract and keyword fields were retrieved and examined for tree deposition. For fungi, 18 specialized and 9 non-specialized journals were individually reviewed (Tables S1 & S2), and each study was examined for tree file and alignment deposition. Phylogenetic literature for seed plants was obtained by searching the angiosperm phylogeny website ([1]; <http://www.mobot.org/mobot/research/apweb/>). Most clades (e.g., families, orders) on this website have a phylogeny section in which the most definitive references for a given clade are discussed. We used publications from the cited references as the basis for our literature search, and examined studies from 88 specialized and 8 non-specialized journals (Tables S1-S3). For seed plants, only publications that presented at least some original sequence data (i.e., alignments were not based solely on data from GenBank or TreeBASE) were examined. For the microbial lineages, TreeBASE, Dryad, and Web of Knowledge were searched for tree file deposition. For all examined clades, the search results were filtered to remove those studies previously identified within the examined two public repositories. This database was used to assess the effectiveness of data retrieval.

From the list of publications above that did not have data publicly archived and available we randomly selected 100 studies (50 from specialized and 50 from non-specialized journals) from each of the four focus groups [1) animals; 2) fungi; 3) spermatophytes; and 4) microbial eukaryotes, archaea, and bacteria], and formally contacted the corresponding authors with an email requesting trees and alignments for the Open Tree of Life project. Our emails stated the purpose of the survey and outlined the relevance of their contributions. An additional reminder was sent to those authors who did not respond to the first email after two weeks. The final statistics for author’s response were estimated one week from the second request email and categorized according to the criteria showed in Table S4.

Next, for each taxonomic group [1) animals; 2) fungi; 3) spermatophytes; and 4) microbial eukaryotes, archaea, and bacteria], we randomly selected 100 studies [50 from specialized journals (see Table S1) and 50 from non-specialized journals (see Tables S2 & S3)] that did have data publicly available on TreeBASE. In order to assess the quality of the deposited data we asked the following questions: 1) Are all the phylogenetic trees presented in the publication deposited? 2) Does the algorithm used in the deposited tree file correspond to the algorithm used to build the figure presented in the publication? 3) Do the tips in the deposited tree file correspond to the tip labels shown in the figure? 4) Does the deposited tree file have branch lengths? 5) Does the deposited tree file have bootstrap support/posterior probability values? 6) Are the sequences produced by the study deposited in GenBank? and 7) Does the TreeBASE ID in the paper match with the one in the database? Unfortunately, within the TreeBASE database there were not 100 publications involving microbial eukaryotes, archaea, and bacteria, thus only 44 (27 non-specialized and 17 specialized) were selected for these taxonomic groups. For seed plants, only 16 of the TreeBASE files examined came from non-specialized journals, while 84 were from specialized journals. In total, we examined TreeBASE deposits from 344 publications, 143 from non-specialized journals and 201 from specialized journals.

Finally, we investigated the availability of additional data sources that without proper public storage can preclude the reproducibility of phylogenetic studies. For this purpose the popular evolutionary analysis package BEAST (Bayesian Evolutionary Analysis Sampling Trees; [2]) offers an excellent framework. This software has been extensively used (4060 citations as of 06-20-2013) since its release because it provides considerable flexibility to specify evolutionary models. All parameters can be modeled given informative priors and easily be stored in an xml file extension, which is requisite to replicate a study. To examine BEAST xml file deposition we randomly chose 100 studies published within the past five years and searched the article for file links, Dryad for file submission, and the online version of the article for supplemental data.

**Literature Cited**

1. Drummond AJ, & Rambaut, A (2007) BEAST: Bayesian evolutionary analysis by sampling trees. BMC Evol Biol 7: 214.
2. Stevens PF (2012) Angiosperm Phylogeny Website. Version 12, July 2012.
