# Supplementary material for: Lost Branches on the Tree of Life
Source: PLoS Biol. 2013 Sep 3;11(9):e1001636. doi: 10.1371/journal.pbio.1001636 (PMC3760775; doi:10.1371/journal.pbio.1001636)
Supplement: Table S4 — Corresponding author's response to emails (2) requesting alignments and trees from previously published study. Number in parenthesis subtending organismal group represents number of authors contacted. (DOCX) [file pbio.1001636.s005.docx]

Table S4. Corresponding author’s response to emails (2) requesting alignments and trees from previously published study. Number in parenthesis subtending organismal group represents number of authors contacted.

|  | FUNGI (100) | SPERMATOPHYTA  (100) | ANIMALIA (100) | UNICELLULAR* (75) |
| --- | --- | --- | --- | --- |
| Corresponding author’s email invalid/new email not found | 9% | 5% | 7% | 0% |
| No response | 45% | 54% | 68% | 80% |
| Data no longer accessible | 7% | 6% | 3% | 7% |
| Author promises to send data (but has not done so) | 4% | 9% | 5% | 0% |
| Author promises to upload data into TreeBASE/Dryad (but has not) | 5% | 0% | 3% | 0% |
| Author uploaded tree into TreeBASE | 1% | 0% | 2% | 0% |
| Author sent alignments not trees | 6% | 0% | 4% | 3% |
| Author sent tree files and alignment | 23% | 22% | 8% | 11% |
| Author is too busy - will not send data | 0% | 4% | 0% | 0% |

*Unicellular includes microbial eukaryotes, archaea, and bacteria
